# Intrinsic nanostructure induced ultralow thermal conductivity yields enhanced thermoelectric performance in Zintl phase Eu_2_ZnSb_2_

**DOI:** 10.1038/s41467-021-25483-w

**Published:** 2021-09-29

**Authors:** Chen Chen, Zhenzhen Feng, Honghao Yao, Feng Cao, Bing-Hua Lei, Yumei Wang, Yue Chen, David J. Singh, Qian Zhang

**Affiliations:** 1 School of Materials Science and Engineering and Institute of Materials Genome & Big Data, Harbin Institute of Technology, Shenzhen, China; 2 Department of Mechanical Engineering, The University of Hong Kong, Hong Kong SAR, China; 3 Institute for Computational Materials Science, School of Physics and Electronics, Henan University, Kaifeng, China; 4 School of Science, Harbin Institute of Technology, Shenzhen, China; 5 Department of Physics and Astronomy, University of Missouri, Columbia, MO USA; 6 Beijing National Laboratory for Condensed Matter Physics, Institute of Physics, Chinese Academy of Science, Beijing, China; 7 Department of Chemistry, University of Missouri, Columbia, MO USA

**Keywords:** Energy, Thermoelectrics

## Abstract

The Zintl thermoelectric phase Eu_2_ZnSb_2_ has a remarkable combination of high mobility and low thermal conductivity that leads to good thermoelectric performance. The key feature of this compound is a crystal structure that has a Zn-site with a 50% occupancy. Here we use comparison of experimental thermal conductivity measurements and first principles thermal conductivity calculations to characterize the thermal conductivity reduction. We find that partial ordering, characterized by local order, but Zn-site disorder on longer scales, leads to an intrinsic nanostructuring induced reduction in thermal conductivity, while retaining electron mobility. This provides a direction for identifying Zintl compounds with ultralow lattice thermal conductivity and good electrical conductivity.

## Abstract (web summary)

Understanding mechanisms for obtaining low thermal conductivity without degraded electrical properties remains a challenge. Here, the authors reveal a mechanism for thermal conductivity reduction in a Zintl phase thermoelectric based on length scales for disorder in Eu_2_ZnSb_2_.

## Introduction

Understanding heat conduction in solids is an ongoing challenge in condensed matter physics with important practical implications^1–7^. This is particularly so in low thermal conductivity materials of importance for thermoelectrics and in thermal barrier materials, where for example perturbative pictures based on phonons and phonon scattering may breakdown due to strong scattering from disorder and/or strong anharmonicity. Moreover, identifying mechanisms that allow reduction of thermal conductivity without commensurate reductions in electrical conductivity remains a central problem in thermoelectrics research^5,8–12^. It has been addressed by the introduction of rattling modes associated with weakly bound atoms^12–14^, alloy scattering^15^, disorder, nanostructuring^16^, anharmonic bonds associated with lone pairs^17–20^, and the selection of materials with complex Zintl structures^21–23^ or soft anharmonic phonons^24,25^, for example near structural phase transitions^26^. High thermoelectric performance has been achieved in materials with intrinsic low thermal conductivity including Cu_2_Se^27^, SnSe^28^, MgAgSb^29^, Yb_14_MnSb_11_^30^, and Ag_9_GaSe_6_^31^. These results illustrate the importance of searching for materials with low thermal conductivity to find thermoelectric compositions.

However, all approaches for achieving low thermal conductivity have limitations. In particular, phonon-based approaches generally yield very low thermal conductivities only at high temperatures, where the effects of anharmonic phonon scattering are strongest, while disorder-based approaches typically lower the thermal conductivity, but also in general strongly affect electronic conductivity so that only certain amounts and types of disorder can be used. Thus, understanding mechanisms for obtaining low thermal conductivity without degraded electrical properties remains an important challenge.

Here we demonstrate a direction, where a partially filled site behaves as if it is ordered for electrons but disordered for heat conduction. This can be understood in terms of the different length scales for phonon and electron scattering and represents an intrinsic nanostructure induced thermal conductivity reduction analogous to observed enhancements in thermoelectric performance in certain artificially nanostructured bulk thermoelectrics^16,32^.

Zintl-phase Eu_2_ZnSb_2_ exhibits an intrinsically ultralow thermal conductivity combined with high electrical mobility, leading to a high thermoelectric figure of merit, *ZT*~1.0 at high temperature^33,34^. Crystal structure refinement yields a hexagonal structure with partial occupancy of the Zn site^35,36^. This partial occupancy implies disorder. However, locally a reasonably high degree of order is expected. This is based on electron microscopy and also on electronic structure calculations that show the band gap formation is highly sensitive to Zn ordering^35^. The compound has a carrier mobility of 50 cm^2^ V^−1^ s^−1^ or above at ambient temperature, which is a value that is not generally consistent with the high degree of carrier scattering that would be expected with extensive disorder on a site that contains a mixture of cations and vacancies and which is intimately involved in the band structure formation. The combination of high mobility and very low thermal conductivity is very unusual, as is the fact that the low thermal conductivity exists not only at very high temperature, but at ambient temperature as well. Here we combine experiment and analysis based on model behavior and first principles calculations, including anharmonic phonon scattering to understand the low thermal conductivity of this material.

## Results and discussion

### Crystallographic structure properties

**Fig. 1:**
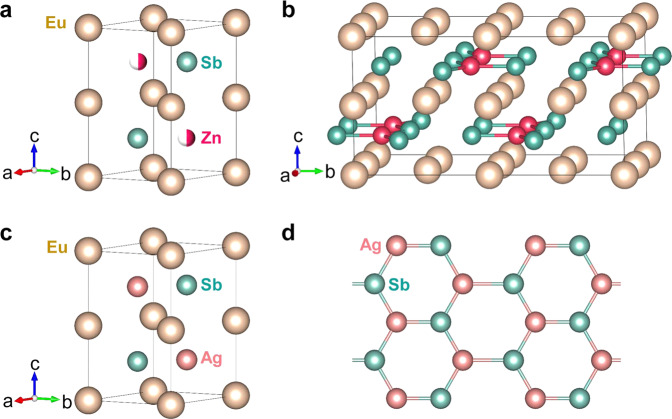
Crystal structures. **a** Crystal structure of Eu_2_ZnSb_2_. The golden, green, and red spheres represent the europium, antimony, and zinc, respectively. **b** Zig-zag-type crystal structure of Eu_2_ZnSb_2_. **c** Crystal structure of EuAgSb. **d** Anionic AgSb layer in EuAgSb. See also Supplementary Material, Fig. S2.

As shown in Fig. 1, Zintl-phase Eu_2_ZnSb_2_ and EuAgSb are based on the same hexagonal crystal structure (space group: *P*6_3_/*mmc*). In the case of Eu_2_ZnSb_2_ there are 50% vacancies on the Zn site. This is understood in terms of the Zintl concept as providing electronic charge balance as monovalent Ag^+^ is replaced by divalent Zn^2+^. Thus the vacancies are needed for obtaining a semiconducting gap. Such a semiconducting gap is observed and is essential for thermoelectric performance. In addition, the stoichiometry is supported by X-ray diffraction Rietveld refinement^35^. In contrast, the site is fully occupied by Ag atoms in EuAgSb^36,37^. However, as noted, electronic structure calculations show that the details of the electronic structure near the band edges are dependent on the specific ordering of the Zn vacancies. In other words, there is an important interplay between Zn vacancy order and the electronic structure near the band edge^35^. This implies strong electron scattering, and resulting low mobility, if the Zn atoms are strongly disordered on length scales comparable to electron mean free paths. Thus, although the exact structure is as yet unknown, two things are known, specifically that the Zn site in the average structure is at 50% occupancy and that there must be a high degree of Zn order at least locally. This is supported by imaging data shown in Supplementary materials, Fig. S1.

### Thermoelectric properties

**Fig. 2:**
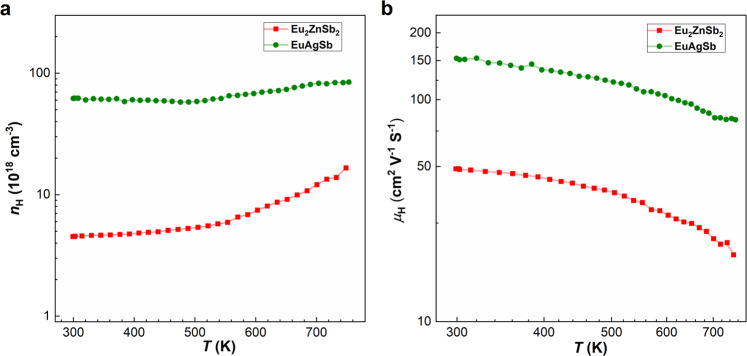
Temperature-dependent Hall data. **a** Carrier concentration and **b** Hall mobility of Eu_2_ZnSb_2_ and EuAgSb^33^.

**Fig. 3:**
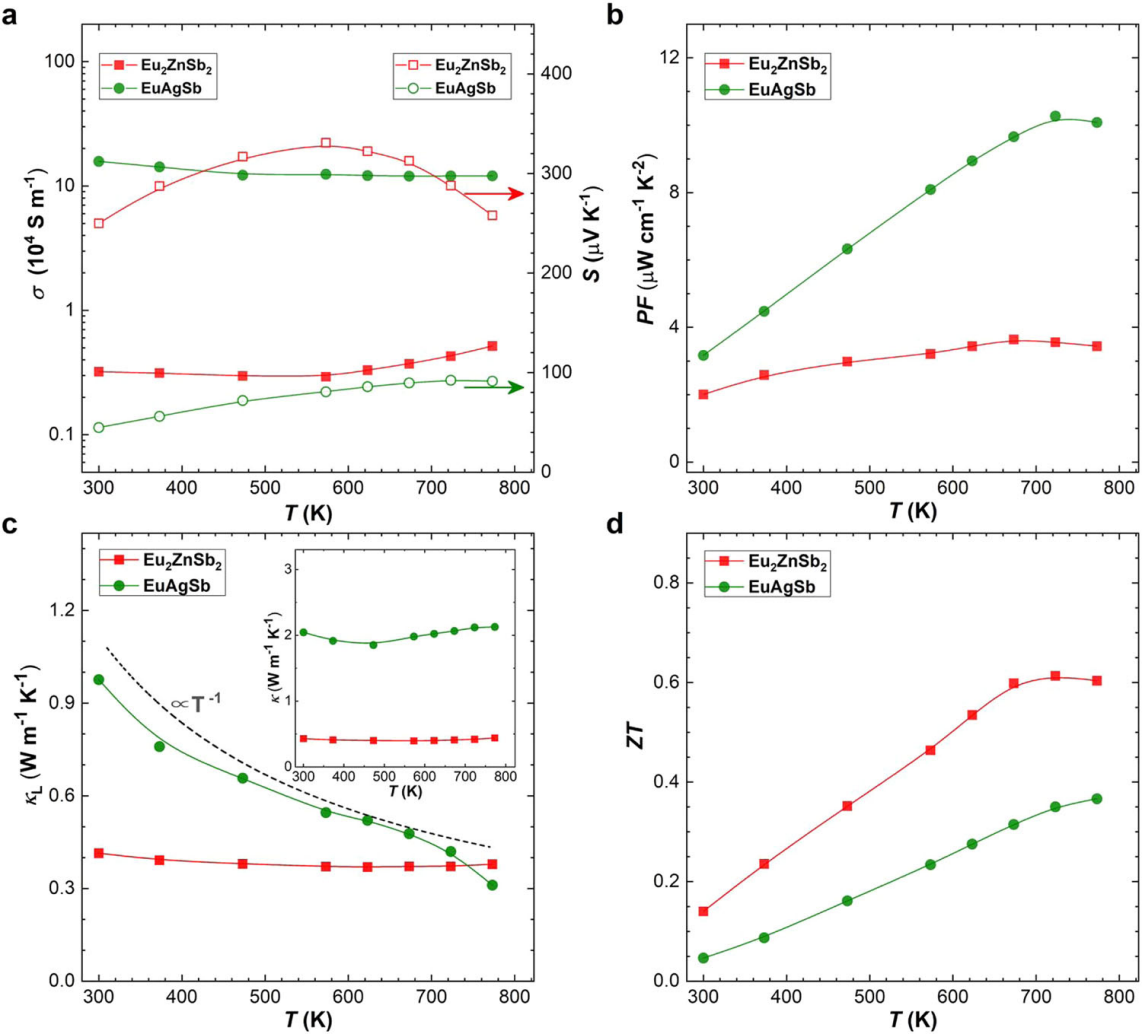
Temperature-dependent transport properties. **a** Electrical conductivity and Seebeck coefficient. **b** Power factor. **c** Thermal conductivity and lattice thermal conductivity. **d** *ZT* values of Eu_2_ZnSb_2_ and EuAgSb^33^.

Thermoelectric samples of EuAgSb have both higher carrier concentration and carrier mobility^38^, as compared with Eu_2_ZnSb_2_ (see Fig. 2). The high carrier mobility of EuAgSb is understood as mainly a consequence of the Ag–Sb framework, which is fully occupied and beneficial to the carrier transport. Temperature-dependent thermoelectric properties are shown in Fig. 3. Compared with Eu_2_ZnSb_2_, the higher carrier concentration of EuAgSb leads to higher electrical conductivity and smaller Seebeck coefficient, as usual. The maximum power factor of Eu_2_ZnSb_2_ is only ~3.5 μW cm^−1^ K^−2^. This compares with the higher maximum power factor of EuAgSb, which can be greater than ~10 μW cm^−1^ K^−2^.

However, the thermal conductivity of EuAgSb is much higher than that of Eu_2_ZnSb_2_. This total thermal conductivity includes both the lattice and electronic contributions. In order to obtain the lattice thermal conductivity, *κ*_L_, the electronic contribution, *κ*_c_ was subtracted from the measured total thermal conductivity, *κ*. The value of *κ*_c_ was estimated based on the Wiedemann–Franz relationship (*κ*_*c*_ = *L*σ*T*). Here *L* is the single parabolic band (SPB) model Lorenz number^39^. This SPB model captures the reduction in *L* near band edges of thermoelectric materials, and is based on the Seebeck coefficient that can be obtained experimentally. The agreement of this model with full Boltzmann transport calculations is generally reasonable, and going beyond it requires the detailed band structure and scattering models, which would depend on the details of the presently unknown Zn vacancy ordering^40^. The electronic contribution is particularly important at higher temperature, where the limitations of this model should be kept in mind.

**Fig. 4:**
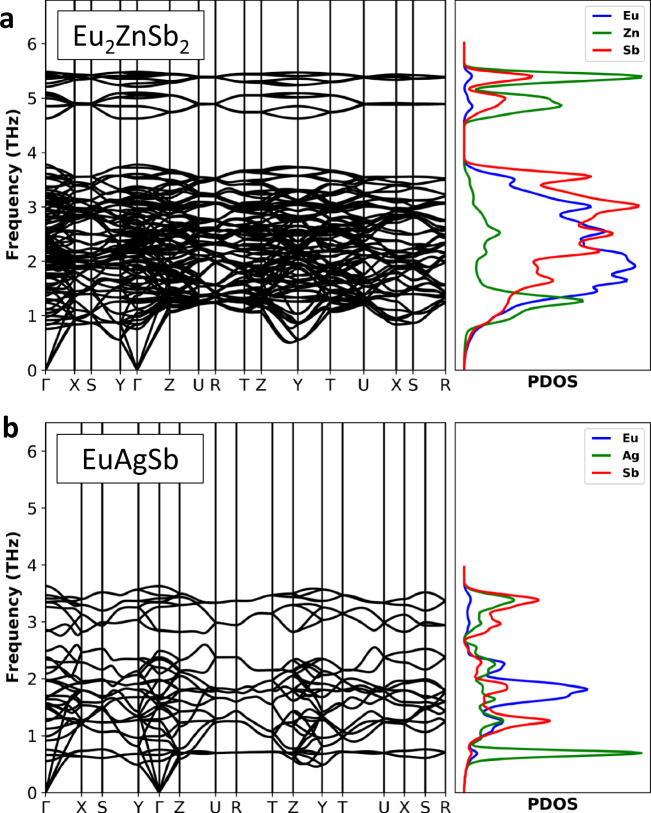
Calculated phonon dispersion relations and phonon density of states. **a** Eu_2_ZnSb_2_ (zig-zag structure). **b** EuAgSb.

Importantly, the lattice thermal conductivity of Eu_2_ZnSb_2_ is close to that of EuAgSb at high temperature, but the value at room temperature is much lower. This leads to a higher *ZT* for Eu_2_ZnSb_2_, especially at lower temperature. Thermoelectric device efficiency is governed by a temperature average, the so-called engineering *ZT*^41^. The lower temperature range, while not enhancing the peak *ZT* is important for the engineering *ZT* and the efficiency of devices that might be made from this material. Understanding this difference in thermal conductivity is therefore key to understanding the high *ZT* of Eu_2_ZnSb_2_. The phonon dispersion relations of Eu_2_ZnSb_2_ and EuAgSb as obtained from density functional theory calculations are shown in Fig. 4. For Eu_2_ZnSb_2_ these were obtained using ordered structures, with zig-zag ordering of Zn vacancies within the ZnSb_2_ plane. As mentioned, the electronic structure of Eu_2_ZnSb_2_ at the band edges depends on the details of the Zn ordering, and in fact there are interesting topological aspects to this dependence^35^. However, the bonding and chemistry is expected to be less sensitive to the particular ordering. Importantly, we find that this ordering yields dynamical stability as characterized both by stable phonons and stable ab initio molecular dynamics. This structure has a space group symmetry, *P*mm2, which is lower than the global space group symmetry of Eu_2_ZnSb_2_. It should be noted that the dispersions show flat optic phonon branches in Eu_2_ZnSb_2_ at low energy starting below 1 THz. This leads to a strong peak in the phonon density of states, which has primary Zn character.

Physical properties of Eu_2_ZnSb_2_ and EuAgSb are listed in Table 1. Measured and calculated sound velocities, Debye temperatures, and bulk moduli are given. The measured values are in accord with the calculated properties. This supports the reasonableness of the structural model. There are not clear differences between the two compounds, and specifically none that would imply strong general softness of the lattice of Eu_2_ZnSb_2_ relative to that of EuAgSb, although Eu_2_ZnSb_2_ does have relatively soft acoustic phonons, as characterized by the low longitudinal sound velocity and the corresponding Debye temperature. These properties are comparable to those in EuAgSb, which as noted has a much higher thermal conductivity. Thus, while some of the differences in thermal conductivity could be ascribed to the softer lattice, the effect is not nearly large enough to explain the difference in thermal conductivity.

**Table 1:** Physical properties of Eu_2_ZnSb_2_ and EuAgSb.

		Eu_2_ZnSb_2_	EuAgSb
Space group		P6_3_/mmc	P6_3_/mmc
Lattice thermal conductivity at 300 K	Exp. data	0.42 W m^−1^ K^−1^	0.97 W m^−1^ K^−1^
Density (g cm^−3^)	Exp. data	6.46 g cm^−3^	7.40 g cm^−3^
Longitudinal sound velocity, *v*_l_ (m s^−1^)	Calculated	3389 m s^−1^	3501 m s^−1^
	Exp. data	3190 m s^−1^	3430 m s^−1^
Shear sound velocity, *v*_s_ (m s^−1^)	Calculated	1982 m s^−1^	1894 m s^−1^
	Exp. data	1900 m s^−1^	2000 m s^−1^
Young’s modulus, *E* (GPa)	Exp. data	56.6 GPa	75.5 GPa
Shear modulus, *G* (GPa)	Exp. data	23.1 GPa	30.4 GPa
Poisson ratio, *v*_p_	Exp. data	0.23	0.24
Debye temperature (K)	Calculated	211 K	224 K
	Exp. data	200 K	218 K
Bulk modulus	Calculated	42.39 GPa	53.6 GPa
	Exp. data	34.6 GPa	48.9 GPa

The experimental data of Young’s modulus, shear modulus, Poisson ratio, Debye temperature, and bulk modulus are estimated using sound velocity.

It is important to note that as temperature increases the experimental thermal conductivity approaches but is not lower than the minimum thermal conductivities allowed within a phonon model^42,43^. The calculated minimum lattice thermal conductivities are 0.42 W/mK and 0.52 W/mK for Eu_2_ZnSb_2_ and EuAgSb, respectively. Thus, the temperature dependence where the thermal conductivity decreases toward a constant value as temperature is increased can be viewed as related to increased phonon scattering as temperature is increased, but the considerably weaker than 1/*T* temperature dependence for Eu_2_ZnSb_2_ suggests additional scattering beyond anharmonic phonon scattering. It is also noteworthy that in addition to the weak temperature dependence of the thermal conductivity in Eu_2_ZnSb_2_ there is a small but noticeable upturn at high temperature. Such upturns can be a consequence of increased thermal conductivity due to transport in localized vibrational modes, as in glassy materials, or bipolar electronic conduction. Such bipolar effects are possible in Eu_2_ZnSb_2_ due to the small ~0.17 eV band gap^33^, and relatively low carrier concentration of ~4.5 × 10^18^ cm^−3^ at ambient temperature. This is also consistent with the fact that, as shown in Fig. 3a, the Seebeck coefficient of Eu_2_ZnSb_2_ decreases with temperature at high temperature, while the conductivity is increasing.

Figure 4 shows the theoretical phonon dispersion curves and the (projected) phonon density of states (PDOS) of Eu_2_ZnSb_2_ and EuAgSb. Both compounds are dynamically stable, as expected. These dispersions show low frequency modes leading to peaks in the PDOS that come from the Zn and Ag. Specifically, there are a set of relatively flat phonon branches leading to a large peak in the PDOS near 1 THz in Eu_2_ZnSb_2_. This is reminiscent of a rattling ion peak, as in clathrates^14^ and filled skutterudites^8,13,44^, where rattling has been associated with very low thermal conductivity. Importantly, this peak has Zn character. This shows that the Zn vibrations can be of particular importance in reducing the thermal conductivity. However, while rattling is expected to lower the thermal conductivity, it does not explain the temperature dependence of the thermal conductivity by itself as discussed below.

**Fig. 5:**
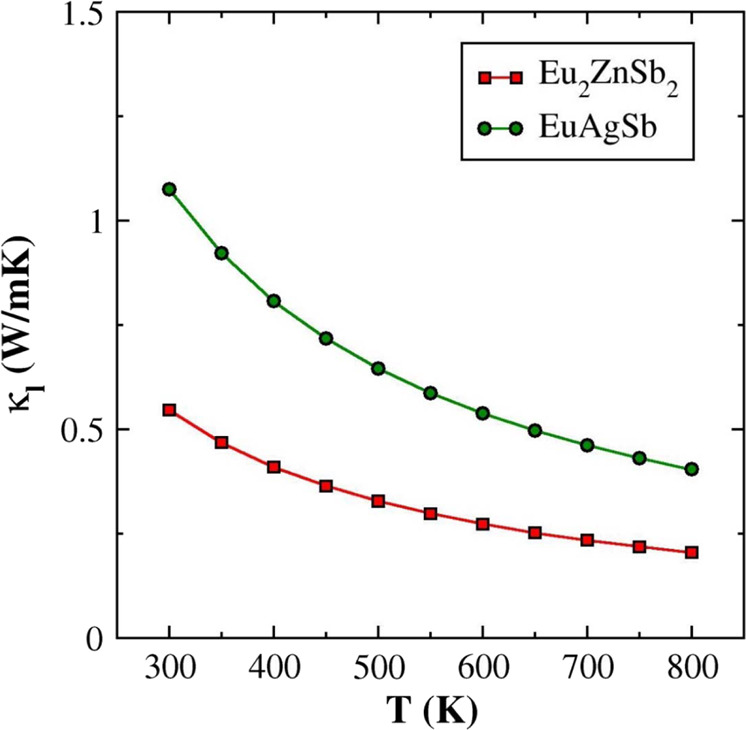
Calculated temperature-dependent lattice thermal conductivities. These are for ordered zig-zag Eu_2_ZnSb_2_ and EuAgSb and are based on anharmonic phonon scattering.

The calculated lattice thermal conductivities of Eu_2_ZnSb_2_ and EuAgSb are shown in Fig. 5, based on anharmonic phonon scattering. As seen, the calculated thermal conductivity of Eu_2_ZnSb_2_ is indeed lower than that of EuAgSb at all temperatures. However, it is higher than the experimental lattice thermal conductivity, in the lower temperature region. The calculated lattice thermal conductivities of Eu_2_ZnSb_2_ are 0.55 W/mK and 0.21 W/mK at 300 K and 800 K, respectively. This may signal a breakdown of the phonon picture at high temperature, though not at ambient temperature. Specifically, at high temperatures or in highly disordered materials, strong scattering leads to localization and a breakdown of the phonon Boltzmann theory in certain materials. This type of breakdown is typically signified by (1) a flat temperature dependence of the thermal conductivity and (2) a thermal conductivity that is higher than the phonon Boltzmann theory due to the contribution to heat conduction of localized incoherent vibrations^2,5,45^. The present calculated result in comparison with experiment indicates that Eu_2_ZnSb_2_ is not in this strong scattering regime at least near ambient temperature though it apparently is at high temperature.

**Fig. 6:**
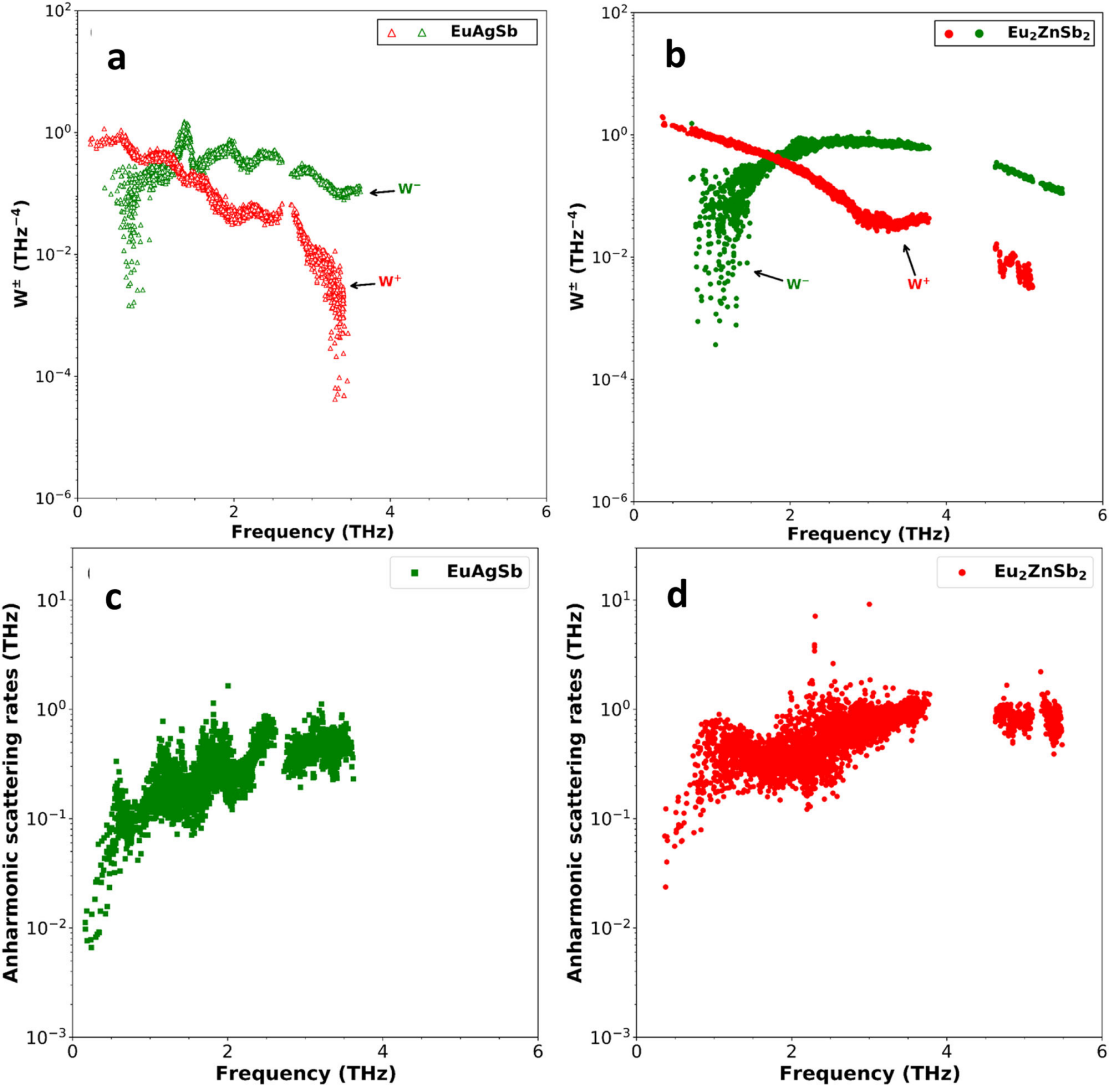
Scattering phase space and scattering rates as a function of phonon frequency. This is the phase space for different phonon modes of **a** zig-zag structure EuAgSb and **b** Eu_2_ZnSb_2_. The +(-) sign represents three-phonon absorption (emission) phase space. The calculated anharmonic scattering rates for **c** EuAgSb and **d** Eu_2_ZnSb_2_.

The very low lattice thermal conductivity of Eu_2_ZnSb_2_ compared to EuAgSb is due to a larger scattering phase space, as shown on a log scale in Fig. 6. This is the case over a wide frequency range starting below 1 THz. The enhancement in scattering phase space is particularly significant in the frequency range between approximately 0.8 THz and 3.5 THz. This reflects in part the Zn-related peak in the PDOS. There is also a significant difference in the anharmonic scattering rates. These are enhanced in Eu_2_ZnSb_2_ relative to EuAgSb, particularly below 2 THz, which is the range that is generally most important for thermal conductivity.

It is noteworthy that the Zn contribution to the PDOS has a two-peak structure in this frequency range, with a sharp peak and then a much broader peak at higher frequency, extending to approximately 3.5 THz. This can be understood as reflecting hybridization between soft Zn vibrations and the “host” lattice Eu–Sb modes. This is in analogy with filled skutterudites where a two-peak feature is also seen in neutron scattering experiments and is associated with thermal conductivity reduction^46^. This provides a general explanation of why Eu_2_ZnSb_2_ has a lower thermal conductivity than EuAgSb, specifically anharmonic scattering involving relatively low-frequency Zn vibrations in analogy with rattling in filled skutterudites. It is interesting that in Eu_2_ZnSb_2_ the rattling atom is the lightest atom in the chemical formula, reflecting weak bonding of the Zn. This underlies the low thermal conductivity near 800 K. However, due to the expected 1/*T* behavior at lower temperature, different from the experimental temperature dependence, the anharmonic phonon scattering picture does not fully explain the lower temperature data.

The experimental data show behavior consistent with anharmonic phonon scattering for EuAgSb with a strong decrease in κ_l_ as a function of temperature. In contrast, the nearly temperature-independent thermal conductivity in the lower temperature regime for Eu_2_ZnSb_2_ is instead consistent with a constant mean free path for the heat carrying phonons. At high temperature this mean free path is constrained by anharmonic phonon scattering, associated with the rattling behavior of the weakly bonded Zn atoms, constrained by the minimum thermal conductivity. This behavior and the difference from the behavior of EuAgSb point to disorder on the Zn site leading to a constrained mean free path as the key mechanism for thermal conductivity reduction at lower temperature. However, since Zn and its ordering play important roles in the band formation, random disorder would be expected to strongly depress the electronic mobility. This would be highly detrimental to the thermoelectric performance, in disagreement with the observed good performance of Eu_2_ZnSb_2_. Thus, there must be disorder on length scales that are of importance for the thermal conductivity and at the same time effective order on the shorter length scales needed for the favorable electronic behavior. This is similar to observations for artificially nanostructured thermoelectrics, where thermal conductivity has been found to be more sensitive to scattering at longer length scales compared to electronic transport. Due to shorter electron mean free paths electronic transport is affected by shorter length scales but can be relatively unaffected by scattering due to structure on longer length scales^16,47,48^.

The Callaway model is useful for analyzing this. Considering the anharmonic phonon scattering due to the rattling like Zn vibrations, we focus on the low-frequency acoustic branches. We then apply the Callaway model to the longitudinal and two transverse acoustic modes, with velocities as in Table 1 and a fixed mean free path, *l*. Above room temperature, the model is *κ*_*l*_ = (*C*_L_*v*_L_*l* + *C*_T_*v*_T_*l*)/3, where *v*_L_ and *v*_T_ are the longitudinal and transverse sound velocities, respectively, and the specific heats are taken as the classical harmonic values of *R* per branch (one longitudinal, two transverse), per mole and the factor of 1/3 is due to the direction averaging. This leads to an estimation of *l* ≈ 150 Å needed for obtaining *κ*_*l*_ = 0.4 W/mK. This is much larger than the lattice parameter and the Zn–Zn spacing in the lattice. This Zn–Zn spacing is the scale that would be provided by random occupancy of the Zn sublattice, so the present result implies that the relevant length scale, *l* is much longer than that which would be provided by fully random Zn occupancy. The effect of the Zn disorder is then to provide phonon scattering similar to a very fine-grained nanostructure, with length scale of approximately 150 Å.

Thus, apparently the Zn atoms in Eu_2_ZnSb_2_ are not fully long-range ordered. When considered at short length scales that are important for the electronic properties the material behaves as if it is ordered, but at length scales of *l* ~ 150 Å disorder is important and scatters phonons. This then allows a separation of length scales, where disorder is important at the longer length scales characteristic of mean free paths of heat-carrying phonons, but is less important at the shorter length scales important for electronic conduction. This is analogous to the effect of artificial nanostructuring where small grain sizes and associated interfaces on length scales important for heat-carrying phonons lower the thermal conductivity, without affecting the electronic transport as strongly^16,32,47^. While the present results do not exclude other mechanisms for thermal conductivity reduction, they do illustrate a way of obtaining phonon–glass, electron–crystal behavior^8,12^, specifically ultralow thermal conductivities over a wide temperature range accompanied by favorable electrical transport.

The intrinsic phonon–phonon interactions with rattling behavior, low bulk modulus, and scattering from disorder on the Zn sublattice lead to low lattice thermal conductivity of Eu_2_ZnSb_2_. This leads to a semiconducting Zintl material with a lattice thermal conductivity close to the minimum thermal conductivity over a very broad temperature range starting at 300 K and extending to high temperature. At the same time, in contrast to the normal situation with a high degree of random disorder, Eu_2_ZnSb_2_ maintains a good mobility leading to high thermoelectric performance. Weakly bonding Zn plays a key role in this. This points the way to a mechanism that can be applied to Zintl-phase materials, particularly the design of systems with disorder on one sublattice, but a local ordering tendency that produces different behavior on different length scales, effectively an intrinsic bulk nanostructured material.

## Methods

### Density functional calculations

Calculations are done using the generalized gradient approximation of Perdew, Burke, and Ernzerhof (PBE-GGA)^49^. Orthorhombic cells corresponding to the zig-zag structure of Eu_2_ZnSb_2_ are used for this compound as well as EuAgSb although EuAgSb has a higher hexagonal symmetry. This is to facilitate comparison of the two compounds. The calculations are done using the projector augmented wave (PAW) method as implemented in the Vienna Ab-initio Simulation Package (VASP) code^50,51^. Geometry optimization is done. A planewave energy cutoff of 500 eV is used along with a 10 × 10 × 10 uniform mesh for Brillouin zone sampling and an energy convergence criterion for self-consistency of 10^−7^ eV. The lattice constants are changed relative to the ideal (disordered) hexagonal structure following ${a}_{0}={a}_{h}\times 2$, ${b}_{0}={b}_{h}\times \sqrt{3}$, ${c}_{0}={c}_{h}$, where ${a}_{0}$, ${b}_{0}$, and ${c}_{0}$ are the unit cell parameters in the orthorhombic crystal system, and ${a}_{h}$, ${b}_{h}$, ${c}_{h}$ are the unit cell parameters in the hexagonal structure. The optimized orthorhombic lattice parameters are 9.18, 15.94, and 8.20 Å for the zig-zag supercell Eu_2_ZnSb_2_. The bulk modulus and related data in Table 1 are from zero K fitting of stress–strain curves. The temperature-dependent effective potential (TDEP) method is used to extract anharmonic force constants^52,53^. This is done to provide a stable well constrained interatomic force constants (IFCs) for the complex crystal structure of these compounds. The TDEP calculation is based on Born–Oppenheimer molecular dynamics with the PAW method at 300 K with Nose thermostat temperature control^54^. A simulation time of 140 ps, with a time step of 1 fs and planewave cutoff of 440 eV are used. An 80 atom zig-zag model supercell of Eu_2_ZnSb_2_ is used for the TDEP calculation for that compound, and a 96 atom supercell is used for EuAgSb. The phonon dispersions are obtained from the effective second-order IFCs using the Phonopy package^55^. The structure is dynamically stable. The thermal conductivities are obtained using the third-order anharmonic IFCs and the ShengBTE package^56^. The convergence with respect to the reciprocal space grid was tested, as shown in Supplemental Material Fig. S3. The temperature dependence of the thermal conductivity is obtained using the temperature-dependent phonon occupations based on the 300 K IFCs.

### Sample preparation and characterization

The samples were prepared by weighing out stoichiometric amounts of silver (Ag, 99.9%, shots), europium (Eu, 99.9%, chunks), zinc (Zn, 99.999%, chunks), and antimony (Sb, 99.999%, shots). These were for the EuAgSb and Eu_2_ZnSb_2_ stoichiometries. These were then loaded into stainless-steel jars. This was done in an argon-filled glove box. The mixtures were ball-milled for 10 h using a high energy ball mill (SPEX 8000M) and subsequently sintered for 2 min using spark plasma sintering under an axial pressure of 60 MPa at 853 K, resulting in dense disks. The density of the resulting Eu_2_ZnSb_2_ sample was 6.46 g/cm^3^, which is approximately 97% of the theoretical density of 6.66 g/cm^3^.

The experimental Young’s modulus, shear modulus, Poisson ratio, Debye temperature, and bulk modulus are approximated from the standard expressions in terms of sound velocities^57^. The longitudinal (*v*_L_) and transverse (*v*_T_) components of the sound velocity were measured using an ultrasonic pulse receiver (Olympus) equipped with an oscilloscope (Tektronix). The Seebeck coefficient (*S*) and electrical conductivity (*σ*) were measured with a ZEM-3 apparatus. The Hall coefficient (*R*_H_) as a function of temperature was measured using the van-der-Pauw technique with a reversible magnetic field of 1.5 T. The Hall mobility, *μ*_H_, and Hall carrier concentration, *n*_H_, were calculated as *μ*_H_ = *σR*_H_ and *n*_H_ = 1/(*eR*_H_), respectively. The thermal conductivity (*к*) was obtained from the thermal diffusivity, specifically *к* = *DαC*_*p*_. Here, *D* is the volumetric density, which was determined by the Archimedes method, *α* is the thermal diffusivity, which was measured using a laser flash apparatus (Netzsch LFA 457), and *C*_*p*_ is the specific heat, which was obtained using a differential scanning calorimetry thermal analyzer (Netzsch DSC 404 F3). We then calculated the lattice thermal conductivity by *κ*_L_ = *κ* – *LσT*, where the Lorenz number (*L*) is from the SPB formula and the measured Seebeck coefficients^39^.

## Supplementary information

Supplementary Information

## Data Availability

Data supporting the conclusions is included in the article. Additional data are available from the corresponding authors upon reasonable request.
