# Supplementary material for: Intrinsic nanostructure induced ultralow thermal conductivity yields enhanced thermoelectric performance in Zintl phase Eu2ZnSb2
Source: Nat Commun. 2021 Sep 29;12:5718. doi: 10.1038/s41467-021-25483-w (PMC8481231; doi:10.1038/s41467-021-25483-w)
Supplement: Supplementary file 1 — Supplementary Information [file 41467_2021_25483_MOESM1_ESM.pdf]

Supplementary Materials:

Intrinsic nanostructure induced ultralow thermal conductivity yields enhanced thermoelectric performance in Zintl phase Eu_2ZnSb_2

Chen Chen,[a,b#] Zhenzhen Feng,[c#] Honghao Yao,[a] Feng Cao,[d] Bing-Hua Lei,[e] , Yumei Wang,[f] Yue Chen,[b*] David J. Singh,[e,g*] and Qian Zhang[a*]

[a] School of Materials Science and Engineering and Institute of Materials Genome & Big Data, Harbin Institute of Technology, Shenzhen 518055, China

[b] Department of Mechanical Engineering, The University of Hong Kong, Pokfulam Road, Hong Kong SAR, China

[c] Institute for Computational Materials Science, School of Physics and Electronics, Henan University, Kaifeng 475004, China

[d] School of Science, Harbin Institute of Technology, Shenzhen 518055, China

[e] Department of Physics and Astronomy, University of Missouri, Columbia MO 65211, USA

[f] Beijing National Laboratory for Condensed Matter Physics, Institute of Physics, Chinese Academy of Science, Beijing 100190, China

[g] Department of Chemistry, University of Missouri, Columbia MO 65211, USA

[*] yuechen@hku.hk; singhdj@missouri.edu; zhangqf@hit.edu.cn

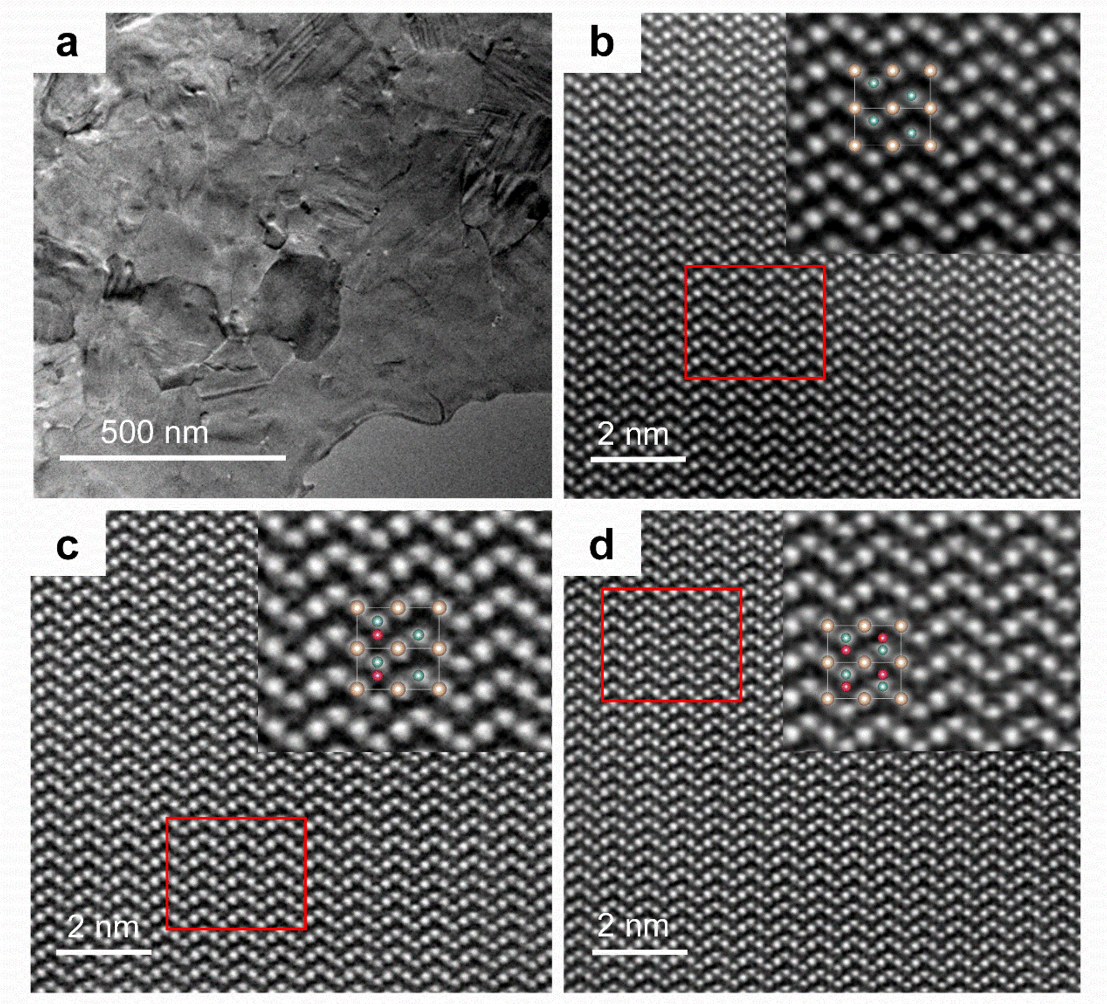

Fig. S1 Imaging data. **a** Low-magnification TEM images of Eu_2ZnSb_2. **b**, **c** and **d** are different HAADF-STEM images along the [100] direction of Eu_2ZnSb_2. Enlarged images are in the insets.

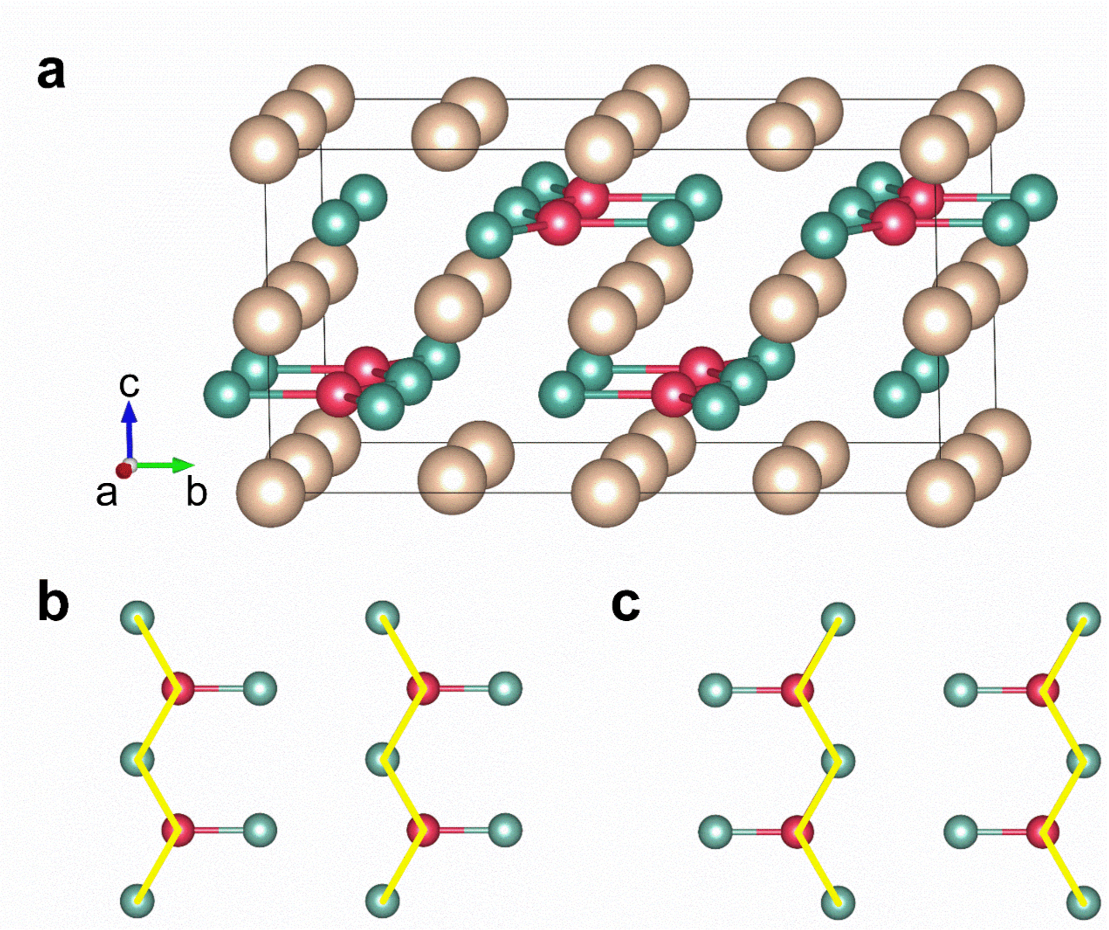

Figure S2. Structural information **a** Crystal structure of zigzag-type Eu_2ZnSb_2. **b** Schematic view of upper-layer $ZnSb_2$ chains. **c** Schematic view of bottom-layer $ZnSb_2$ chains layer. The gold, red, and green spheres represent europium, zinc, and antimony, respectively.

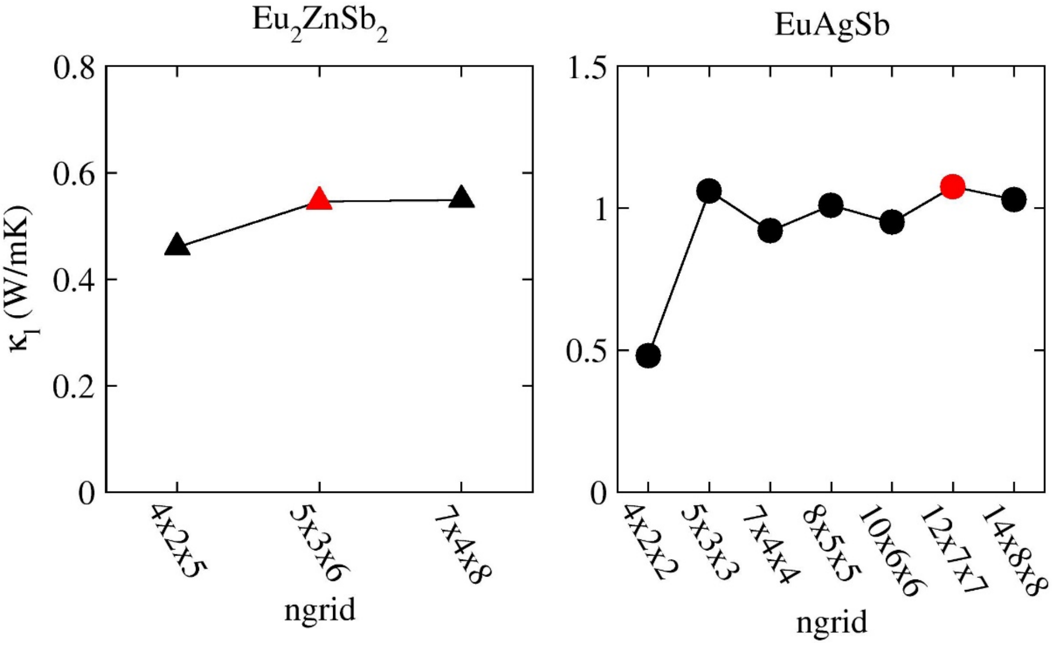

Figure S3. Convergence of the thermal conductivity with respect to grid points in the ShengBTE calculation. The red symbols are the values used for the results shown in the manuscript.
